# 基于改性多壁碳纳米管净化的高效液相色谱-三重四极杆/复合线性离子阱质谱测定南美白对虾中66种农药残留

**DOI:** 10.3724/SP.J.1123.2023.12021

**Published:** 2024-12-08

**Authors:** Ruidong ZHOU, Yupeng WEN, Wendi HUO, Chaoying ZHANG, Huan LIU, Huiwu SUN, Lidong WU, Jincheng LI

**Affiliations:** 1.上海海洋大学水产与生命学院, 上海 201306; 1. College of Fisheries and Life Science, Shanghai Ocean University, Shanghai 201306, China; 2.中国水产科学研究院农业农村部水产品质量安全控制重点实验室, 北京 100141; 2. Key Laboratory of Control of Quality and Safety for Aquatic Products, Ministry of Agriculture and Rural Affairs, Chinese Academy of Fishery Sciences, Beijing 100141, China; 3.北京石油化工学院新材料与化工学院, 北京 102617; 3. School of New Materials and Chemical Engineering, Beijing Institute of Petrochemical Technology, Beijing 102617, China

**Keywords:** 多壁碳纳米管, 高效液相色谱-三重四极杆/复合线性离子阱质谱, 水产品, 农药残留, multi-walled carbon nanotubes (MWCNTs), high performance liquid chromatography-triple quadrupole/linear ion trap mass spectrometry (HPLC-QTRAP-MS/MS), aquatic products, pesticide residues

## Abstract

针对水产品农药残留分析过程中基质干扰强的现状,本研究利用多壁碳纳米管(MWCNTs)和海藻酸钠(SAL)制备了一种MWCNTs-SAL复合净化材料,建立了高效液相色谱-三重四极杆/复合线性离子阱质谱(HPLC-QTRAP-MS/MS)快速测定水产品中66种农药残留的分析方法。采用电喷雾装置将MWCNTs和SAL的复合溶液快速喷入CaCl_2_溶液中进行交联,制备MWCNTs-SAL复合材料。将乙腈作为提取溶剂,基于推杆式震荡型净化技术对提取液进行净化。采用C_18_ RRHD色谱柱(150 mm×3.0 mm, 1.8 μm)对目标化合物进行分离,在多反应监测(MRM)模式下采集质谱数据,使用基质匹配混合标准溶液进行定量分析。实验比较了MWCNTs-SAL填料与商品化填料对南美白对虾提取液的净化效果,并考察了MWCNTs-SAL用量对目标化合物净化效果的影响。实验结果表明,66种农药在0.5~50 μg/L内线性关系良好,相关系数均>0.99,检出限和定量限分别为0.5~1 μg/kg和1~2 μg/kg;在低、中、高3个加标水平下,66种目标化合物的回收率为64.1%~107.3%,日内和日间精密度均小于20.4%。该方法经济快速,简便高效,具有较高的灵敏度和重复性,是水产品中农药残留分析的可行方法。

## Abstract (en)

The complex composition of aquatic products, with high levels of protein and fat, significantly impacts the accuracy of liquid chromatography-mass spectrometry analysis for pesticide residue detection. Hence, developing effective purification materials is essential to mitigate these matrix interferences. To address strong matrix interference in pesticide residue analysis, a composite material, multi-walled carbon nanotubes (MWCNTs)-sodium alginate (SAL) (MWCNTs-SAL), was developed and used as purification material in a push-rod oscillation purification column to establish a rapid method for analyzing 66 pesticide residues in *Litopenaeus vannamei*. The composite solution of MWCNTs and SAL was quickly sprayed into CaCl_2_ solution by electrospray device for crosslinking, and MWCNTs-SAL materials were prepared. The results of scanning electron microscopy image proved the successful preparation of the MWCNTs-SAL. Acetonitrile, as the extraction solvent, was modified with acetic acid to augment the extraction efficiency of acidic pesticides, and anhydrous sodium sulfate was utilized to remove water from the matrix. Push-rod oscillation purification technology was used for purification of the post-centrifugation extract. The target compounds were separated using C_18_ RRHD column (150 mm×3.0 mm, 1.8 μm) and the mobile phases were consisted of 0.2% formic acid aqueous solution (containing 5 mmol/L ammonium acetate) and 0.2% formic acid methanol. The high performance liquid chromatography-triple quadrupole/linear ion trap mass spectrometry (HPLC-QTRAP-MS/MS) was used for separation and determination, and the data was collected through the multiple reaction monitoring (MRM) mode. Due to the different properties of the target compounds, positive and negative ion acquisition scan modes were employed for mass spectrometry analysis. The quantitative analyses of the 66 pesticides were achieved by matrix-matched standard curves. The purification effects of MWCNTs-SAL and commercial sorbents on the extraction of the 66 pesticides were compared. The pretreatment parameters, such as the optimal dosage of MWCNTs-SAL and anhydrous sodium sulfate on the purification effects of the 66 pesticides were also assessed. When using 50 mg MWCNTs-SAL and 100 mg anhydrous sodium sulfate as the purification materials, the purification effect of aquatic products was the best. The 66 pesticides demonstrated good linearities in the range of 0.5-50 μg/L, with correlation coefficients of greater than 0.99. The limits of detection and quantification for the 66 pesticides were 0.5-1 μg/kg and 1-2 μg/kg, respectively. The recoveries of the 66 pesticides under three spiked levels (low, medium, high) were 64.1%-107.3%. The intra- and inter-day precisions were both less than 20.4%. This study provides an economical, quick, efficient, and sensitive approach for pesticide residue detection in aquatic products.

水产品质量安全是现阶段社会关注和政府监管的重点,水产养殖环境中的农药滥用不仅会污染水环境,还会对水产品的质量安全造成威胁^[1]^。水产品中残留的农药可以通过食物链在生物体内富集并对人体健康造成潜在危害。已有研究表明,暴露于某些农药残留与多种急性和慢性疾病相关,包括肝衰竭、肾衰竭以及肿瘤等^[2⇓-4]^。因此,对水产品中残留的农药进行测定对于保障水产品的质量安全具有重要意义。由于水产品基质中的干扰物质含量较高,在农药残留分析过程中,需要采用溶剂萃取、固相萃取、液液萃取等样品前处理方法对水产品基质进行净化,以降低干扰物质对水产品农药残留检测结果的影响^[5,6]^。然而,上述前处理方法存在耗时长和溶剂消耗量大的问题,无法满足农药的快速检测需求。

近年来,随着研究者对复杂体系分析领域的日益关注,多种高效样品制备方法得到了快速发展,如QuEChERS法和快速滤过型净化法^[7]^;其中,快速滤过型净化法凭借其简单、高效以及集净化与固液分离功能于一体的优势,在水产品中化学危害因子的分析方面得到了快速发展,极大地简化了样品处理步骤,提高了分析效率^[8]^。快速滤过型净化法是在QuEChERS法基础上发展而来,其将十八烷基硅烷键合硅胶(C_18_)、乙二胺-*N*-丙基硅烷(PSA)或多壁碳纳米管(multi-walled carbon nanotubes, MWCNTs)等吸附材料填充到净化柱中,形成净化填料层,待样品提取液通过净化填料层后即可完成杂质的去除。快速滤过型净化法中常用的净化柱主要有快速滤过型净化柱(m-PFC)^[9⇓-11]^和推杆式滤过型净化柱^[12]^等。Zhou等^[13]^提出了一种推杆式震荡型净化方法,将C_18_作为净化填料,用于鱼肉组织中7种磺胺类药物和2种镇定剂类药物的残留分析,实现了高通量、半自动化的样品前处理过程。

选择合适的净化材料是获得良好净化效果的关键。与传统净化材料相比,MWCNTs具有比表面积大、机械强度高和热稳定性好等诸多优势,且其可通过离子交换、*π-π*相互作用等方式去除水产品提取液中的脂肪酸和磷脂等干扰物质^[14]^,目前MWCNTs已被广泛用于样品富集和净化领域。王娇等^[15]^以羧基化MWCNTs和C_18_作为复合净化吸附剂,结合液相色谱-串联质谱,建立了中草药中22种三唑类杀菌剂的快速检测方法。涂祥婷等^[16]^将以一定比例混合的C_18_、PSA和MWCNTs作为快速滤过型净化柱的净化材料,建立了鲫鱼体内16种全氟烷基化合物的高效液相色谱-串联质谱检测方法。虽然MWCNTs具有强大的吸附能力,但其分散性差、易团聚、称量困难以及生物兼容性不佳等缺点限制了其在快速滤过型净化柱中的单独应用。海藻酸钠(sodium alginate, SAL)是从褐藻中提取出来的一种天然多糖,具有价格低廉、含量丰富、无毒和可生物降解等优点,且易与Ca^2+^等二价金属阳离子发生交联,形成三维聚合物网络结构,提供更多的吸附位点。

针对上述问题,本研究将具有优良生物兼容性的SAL和具有优异吸附性能的MWCNTs结合,制备了一种经济实用、称量方便、吸附性能优异的MWCNTs-SAL复合净化材料,并基于自制的推杆式震荡型净化柱和高效液相色谱-三重四极杆/复合线性离子阱质谱(HPLC-QTRAP-MS/MS),建立了南美白对虾中66种农药残留的快速定量分析方法。该方法为农药残留检测方法开发、食用水产品农药污染状况调查以及农药残留风险评估等方面提供了理论和技术支持。

## 1 实验部分

### 1.1 仪器、试剂与材料

QTRAP 5500型HPLC-MS/MS系统(美国SCIEX公司); H-2050 R台式高速离心机(湖南湘仪实验室仪器开发有限公司); SB-5200 DTN超声波清洗机(宁波新芝生物科技股份有限公司); MVM-2500多管涡流混合器(上海泰坦科技股份有限公司);固相萃取柱(50 mg/3 mL,上海西木科学仪器有限公司)。

66 种农药混合标准溶液(100 μg/mL)购自上海安谱科技有限公司;乙腈、甲醇(HPLC级)购于美国J. T. Baker公司;无水硫酸钠(纯度≥99.0%)购于中国上海国药化学试剂有限公司;乙酸铵(色谱级)购自美国Thermo Fisher Scientific公司;蒸馏水购自广州屈臣氏食品饮料有限公司;PSA、C_18_、聚苯乙烯-二乙烯基苯填料(HLB)购自北京振翔科技有限公司;海藻酸钠粉购自北京索莱宝科技有限公司;氨基多壁碳纳米管购自南京先锋纳米有限公司。南美白对虾购自北京本地市场。

### 1.2 混合标准工作溶液的配制

用乙腈对66种农药的混合标准溶液进行逐级稀释,得到质量浓度为1 mg/L的混合标准工作溶液,于-20 ℃遮光保存,备用。

### 1.3 MWCNTs-SAL的制备

将2 g SAL溶于100 mL蒸馏水中,磁力搅拌2 h;加入0.5 g MWCNTs继续磁力搅拌6 h,采用便携式电喷雾装置将MWCNTs和SAL的复合溶液喷入含有300 mL CaCl_2_溶液(0.1 mol/L)的烧杯中,得到MWCNTs-SAL水凝胶;将MWCNTs-SAL水凝胶在CaCl_2_溶液中继续浸泡16 h,以确保完全凝胶化;用蒸馏水冲洗上述水凝胶3次,以去除多余的Ca^2+^;随后将复合水凝胶在60 ℃干燥箱中干燥,即得MWCNTs-SAL复合颗粒,备用。

### 1.4 推杆式震荡型净化柱的制备

取一片筛板置于固相萃取柱底部,用打孔器在萃取柱柱管上端打一圆形孔,然后准确称量50 mg MWCNTs-SAL复合颗粒和100 mg无水Na_2_SO_4_,填充至底端带有筛板的3 mL固相萃取柱中,最后向下推动固相萃取柱推杆至加样孔之下,推杆式震荡型净化柱制备完成。

### 1.5 样品制备

#### 1.5.1 提取过程

将南美白对虾去壳,采集肌肉组织,进行均质处理后,于-20 ℃保存。实验前,取出待测虾肌肉样品,恢复至室温,称取2.5 g虾肌肉样品于50 mL离心管中,加入10 mL乙腈,涡旋振荡10 min;随后向上述混合体系中先后加入2 g无水Na_2_SO_4_和100 μL乙酸,涡旋振荡10 min;在8000 r/min下离心5 min,将上层溶液转移至15 mL离心管中,待净化。

#### 1.5.2 净化过程

向上拉动推杆式震荡型净化柱的活塞至加样孔之上,使用移液枪将1 mL待净化提取液加入到柱管中,随后涡旋振荡1 min,推动净化柱活塞,将净化后的提取液转移至进样瓶中,直接上机检测。

### 1.6 分析条件

#### 1.6.1 色谱条件

色谱柱:C_18_ RRHD色谱柱(150 mm×3.0 mm, 1.8 μm);流动相:A相为0.2%甲酸水溶液(含5 mmol/L乙酸铵), B相为0.2%甲酸甲醇溶液,梯度洗脱,洗脱程序见表1。进样量:5.0 μL;柱温箱温度:35 ℃。

**表 1:** 梯度洗脱程序

Time/min	Flow rate/(mL/min)	φ(A)/%	φ(B)/%
0	0.4	95	5
1.0	0.4	95	5
3.0	0.4	80	20
8.0	0.4	50	50
19.0	0.4	5	95
23.0	0.4	5	95
23.1	0.4	95	5
27.0	0.4	95	5

A: 0.2% formic acid aqueous solution (containing 5 mmol/L ammonium acetate); B: 0.2% formic acid methanol.

#### 1.6.2 质谱条件

离子源:电喷雾电离(ESI)源,多反应监测(MRM)模式;喷雾电压:4.5 kV;气帘气压力:0.21 MPa;雾化气压力:0.41 MPa;离子源温度:550.0 ℃;辅助气压力:0.38 MPa。其他质谱参数和保留时间等信息见表2。

**表 2:** 66种农药的保留时间和质谱参数

No.	Compound	Classification	CAS No.	Molecular formula	Retention time/min		Parent ion (*m/z*)		Product ions (*m/z*)	DP/ V	CEs/ eV
1	methamidophos (甲胺磷)	organophosphates	10265-92-6	C_2_H_8_NO_2_PS	4.91		142.0		94.0^*^, 125.0	57	19, 18
2	acephate (乙酰甲胺磷)	organophosphates	30560-19-1	C_4_H_10_NO_3_PS	5.75		184.0		143.0^*^, 125.0	50	12, 25
3	omethoate (氧化乐果)	organophosphates	1113-02-6	C_5_H_12_NO_4_PS	6.34		214.0		183.0^*^, 109.0	60	16, 36
4	propamocarb (霜霉威)	carbamates	24579-73-5	C_9_H_20_N_2_O_2_	6.42		189.2		102.1^*^, 74.1	70	24, 35
5	aldicarb-sulfoxide (涕灭威亚砜)	carbamates	1646-87-3	C_7_H_14_N_2_O_3_S	6.72		207.1		132.0^*^, 89.0	55	9, 20
6	aldicarb-sulfone (涕灭威砜)	carbamates	1646-8-4	C_7_H_14_N_2_O_4_S	7.14		240.1		148.0^*^, 166.1	30	17, 16
7	carbendazim (多菌灵)	imidazoles	10605-21-7	C_9_H_9_N_3_O_2_	7.92		192.1		160.1^*^, 132.1	80	25, 41
8	methomyl (灭多威)	carbamates	16752-7-5	C_5_H_10_N_2_O_2_S	7.93		163.1		88.0^*^, 106.0	38	12, 14
9	thiamethoxam (噻虫嗪)	neonicotinoids	153719-23-4	C_8_H_10_ClN_5_O_3_S	8.15		292.0		211.1^*^, 181.1	30	16, 30
10	imidacloprid (吡虫啉)	neonicotinoids	138261-41-3	C_9_H_10_ClN_5_O_2_	9.32		256.1		209.1^*^, 175.1	45	22, 27
11	clothianidin (可尼丁)	neonicotinoids	210880-92-5	C_6_H_8_ClN_5_O_2_S	9.46		250.0		132.0^*^, 169.1	35	21, 17
12	carbofuran-3-hydroxy (3-羟基克百威)	carbamates	16655-82-6	C_12_H_15_NO_4_	10.03		238.1		181.1^*^, 163.1	70	16, 18
13	acetamiprid (啶虫脒)	neonicotinoids	135410-20-7	C_10_H_11_ClN_4_	10.05		223.1		126.0^*^, 99.0	65	28, 60
14	dimethoate (乐果)	organophosphates	60-51-5	C_5_H_12_NO_3_PS_2_	10.07		230.0		125.0^*^, 199.0	56	29, 13
15	aldicarb (涕灭威)	carbamates	116-06-3	C_7_H_14_N_2_O_2_S	11.66		208.1		116.1^*^, 89.0	20	11, 25
16	dichlorvos (敌敌畏)	organophosphates	62-73-7	C_4_H_7_Cl_2_O_4_P		12.77		221.0	109.0^*^, 127.0	70	23, 27
17	thiophanatemethyl (甲基托布津)	imidazoles	23564-05-8	C_12_H_14_N_4_O_4_S_2_		12.80		343.1	151.0^*^, 311.0	60	26, 15
18	carbofuran (克百威)	carbamates	1563-6-2	C_12_H_15_NO_3_		13.06		222.1	165.1^*^, 123.0	70	16, 29
19	fenthion-sulfoxide (倍硫磷亚砜)	organophosphates	3761-41-9	C_10_H_15_O_4_PS_2_		13.32		295.0	280.0^*^, 109.0	90	25, 45
20	fenthion-sulfone (倍硫磷砜)	organophosphates	3761-42-0	C_10_H_15_O_5_PS_2_		13.69		311.0	124.8^*^, 278.9	100	25, 23
21	phorate-sulfoxide (甲拌磷亚砜)	organophosphates	2588-3-6	C_7_H_17_O_3_PS_3_		14.28		277.0	199.0^*^, 153.0	25	13, 19
22	phorate-sulfone (甲拌磷砜)	organophosphates	2588-4-7	C_7_H_17_O_4_PS_3_		14.50		293.0	97.0^*^, 115.0	65	50, 35
23	isoprocarb (异丙威)	carbamates	2631-40-5	C_11_H_15_NO_2_		14.59		194.1	95.1^*^, 137.1	57	19, 12
24	pyrimethanil (嘧霉胺)	phenylpyrazoles	53112-28-0	C_12_H_13_N_3_		14.93		200.1	183.1^*^, 168.1	30	33, 40
25	forchlorfenuron (氯吡苯脲)	-	68157-60-8	C_12_H_10_ClN_3_O		14.96		248.1	129.0^*^, 93.0	50	23, 47
26	methidathion (杀扑磷)	organophosphates	950-37-8	C_6_H_11_N_2_O_4_PS_3_		15.31		303.0	145.0^*^, 85.0	59	13, 28
27	azoxystrobin (嘧菌酯)	strobilurin fungicides	131860-3-8	C_22_H_17_N_3_O_5_		15.85		404.1	372.1^*^, 344.1	80	20, 34
28	dimethomorph (烯酰吗啉)	amides fungicides	110488-70-5	C_21_H_22_ClNO_4_		16.63		388.1	301.1^*^, 165.1	105	29, 43
29	malathion (马拉硫磷)	organophosphates	121-75-5	C_10_H_19_O_6_PS_2_		16.67		331.0	127.0^*^, 99.0	70	16, 32
30	myclobutanil (腈菌唑)	triazoles	88671-89-0	C_15_H_17_ClN_4_		16.85		289.1	70.0^*^, 125.0	80	35, 46
31	triazophos (三唑磷)	organophosphates	24017-47-8	C_12_H_16_N_3_O_3_PS		17.02		314.1	162.1^*^, 119.1	80	24, 50
32	isazofos (氯唑磷)	organophosphates	42509-80-8	C_9_H_17_ClN_3_O_3_PS		17.10		316.0	164.0^*^, 122.0	70	23, 35
33	fipronil-desulfinyl (氟甲腈)	benzoylureas	205650-65-3	C_12_H_4_Cl_2_F_6_N_4_		17.45		387.0	351.0^*^, 282.0	-30	-19, -47
34	epoxiconazole (氟环唑)	triazoles	133855-98-8	C_17_H_13_ClFN_3_O		17.56		330.1	121.0^*^, 101.0	85	55, 70
35	ethoprophos (灭线磷)	organophosphates	13194-48-4	C_8_H_19_O_2_PS_2_		17.59		243.1	130.9^*^, 97.0	67	26, 43
36	fenbuconazole (腈苯唑)	triazoles	114369-43-6	C_19_H_17_ClN_4_		17.63		337.1	125.0^*^, 70.0	95	42, 43
37	fipronil (氟虫腈)	benzoylureas	120068-37-3	C_12_H_4_Cl_2_F_6_N_4_OS		17.77		434.9	330.0^*^, 250.0	-25	-24, -38
38	flusilazole (氟硅唑)	triazoles	85509-19-9	C_16_H_15_F_2_N_3_Si		17.79		316.1	247.1^*^, 165.1	50	26, 37
39	iprodione (异菌脲)	oxadiazines	36734-19-7	C_13_H_13_Cl_2_N_3_O_3_		17.82		330.1	245.0^*^, 288.0	30	20, 18
40	fipronil-sulfide (氟虫腈亚砜)	benzoylureas	120067-83-6	C_12_H_4_Cl_2_F_6_N_4_S		17.96		418.9	383.0^*^, 262.0	-20	-22, -35
41	prochloraz (咪鲜胺)	imidazoles	67747-09-5	C_15_H_16_Cl_3_N_3_O_2_		18.23		376.2	308.0^*^, 266.0	20	15, 22
42	isofenphosmethyl (甲基异柳磷)	organophosphates	99675-03-3	C_14_H_22_NO_4_PS		18.24		332.1	231.0^*^, 121.0	20	19, 43
43	isocarbophos (水胺硫磷)	organophosphates	24353-61-5	C_11_H_16_NO_4_PS		18.24		231.0	121.0^*^, 109.0	100	26, 38
44	fipronil-sulfone (磺基氟虫腈)	benzoylureas	120068-36-2	C_12_H_4_Cl_2_F_6_N_4_O_2_S		18.27		450.9	414.9^*^, 282.0	-28	-26, -38
45	tebuconazole (戊唑醇)	triazoles	107534-96-3	C_16_H_22_ClN_3_O		18.37		308.2	70.0^*^, 125.0	95	49, 47
46	fenthion (倍硫磷)	organophosphates	55-38-9	C_10_H_15_O_3_PS_2_		18.38		279.0	247.0^*^, 169.0	78	18, 23
47	famoxadone (噁唑菌酮)	oxazoles	131807-57-3	C_22_H_18_N_2_O_4_		18.44		392.2	331.1^*^, 238.1	45	12, 24
48	phoxim (辛硫磷)	organophosphates	14816-18-3	C_12_H_15_N_2_O_3_PS		18.70		299.1	129.0^*^, 153.0	55	18, 10
49	pyraclostrobin (百克敏)	strobilurin fungicides	175013-18-0	C_19_H_18_ClN_3_O_4_		18.71		388.1	194.1^*^, 163.1	50	18, 36
50	hexaconazole (己唑醇)	triazoles	79983-71-4	C_14_H_17_Cl_2_N_3_O		18.76		314.1	70.0^*^, 159.0	85	45, 40
51	phorate (甲拌磷)	organophosphates	298-02-2	C_7_H_17_O_2_PS_3_		18.96		261.0	75.0^*^, 47.0	51	21, 53
52	difenoconazole (苯醚甲环唑)	triazoles	119446-68-3	C_19_H_17_Cl_2_N_3_O_3_		19.15		406.1	251.0^*^, 337.0	105	35, 24
53	(E)-diniconazole ((E)-烯唑醇)	triazoles	83657-24-3	C_15_H_17_Cl_2_N_3_O		19.15		326.1	70.0^*^, 159.0	105	60, 40
54	emamectin benzoate (甲氨基阿维菌素苯甲酸盐)	macrocyclic lactones	155569-91-8	C_56_H_81_NO_15_		19.25		886.5	158.1^*^, 82.1	50	41, 110
55	trifloxystrobin (肟菌酯)	strobilurin fungicides	141517-21-7	C_20_H_19_F_3_N_2_O_4_		19.33		409.1	186.1^*^, 145.0	40	23, 63
56	tolfenpyrad (唑虫酰胺)	amides fungicides	129558-76-5	C_21_H_22_ClN_3_O_2_		20.18		384.1	197.1^*^, 171.0	40	35, 33
57	fenpropathrin (甲氰菊酯)	pyrethroids	39515-41-8	C_22_H_23_NO_3_		20.60		350.2	97.1^*^, 125.1	85	46, 23
58	chlorpyrifos (毒死蜱)	organophosphates	2921-8-2	C_9_H_11_Cl_3_NO_3_PS		20.61		349.9	197.9^*^, 97.0	75	28, 45
59	pendimethalin (二甲戊乐灵)	anilides fungicides	40487-42-1	C_13_H_19_N_3_O_4_		20.72		282.1	212.1^*^, 194.1	40	15, 28
60	etoxazole (乙螨唑)	-	153233-91-1	C_21_H_23_F_2_NO_2_		20.84		360.2	141.0^*^, 304.1	80	42, 25
61	propargite (克螨特)	organosulfurs	2312-35-8	C_19_H_26_O_4_S		20.86		368.2	231.2^*^, 175.2	20	14, 21
62	(E)-fenpyroximate ((E)-唑螨酯)	benzoylphenylureas	134098-61-6	C_24_H_27_N_3_O_4_		21.18		422.2	135.0^*^, 336.1	90	43, 23
63	esfenvalerate (顺式氰戊菊酯)	carbamates	66230-04-4	C_25_H_22_ClNO_3_		21.29		523.0	280.9^*^, 506.0	55	23, 16
64	fenvalerate (氰戊菊酯)	pyrethroids	51630-58-1	C_25_H_22_ClNO_3_		21.41		437.2	167.1^*^, 125.0	30	19, 59
65	pyridaben (哒螨灵)	-	96489-71-3	C_19_H_25_ClN_2_OS		21.64		365.1	309.1^*^, 147.1	77	17, 34
66	bifenthrin (联苯菊酯)	pyrethroids	82657-04-3	C_23_H_22_ClF_3_O_2_		22.65		440.2	181.1^*^, 166.1	40	22, 58

DP: declustering potential; CE: collision energy; * quantitative ion; -: unclassified.

## 2 结果与讨论

### 2.1 仪器分析条件的优化

由于本实验中66种目标农药的酸碱性质不同,采用正、负离子同时扫描的模式进行质谱分析。首先,对66种目标化合物进行全扫描,确认母离子;之后,对目标化合物的母离子进行产物离子扫描,筛选特征碎片离子,选择响应强度最高的两个特征碎片离子分别作为定量离子和定性离子;最后,对定量和定性离子的去簇电压和碰撞能量等质谱参数进行优化,确定最优质谱条件,详见表2。

在采用0.2%甲酸水溶液(含5 mmol/L乙酸铵)和0.2%甲酸甲醇溶液作为流动相的条件下,大部分目标化合物的色谱峰具有较高的响应强度且色谱峰分离度良好。在最佳仪器分析条件下,66种目标化合物的总离子流色谱图见图1。

**图 1:**
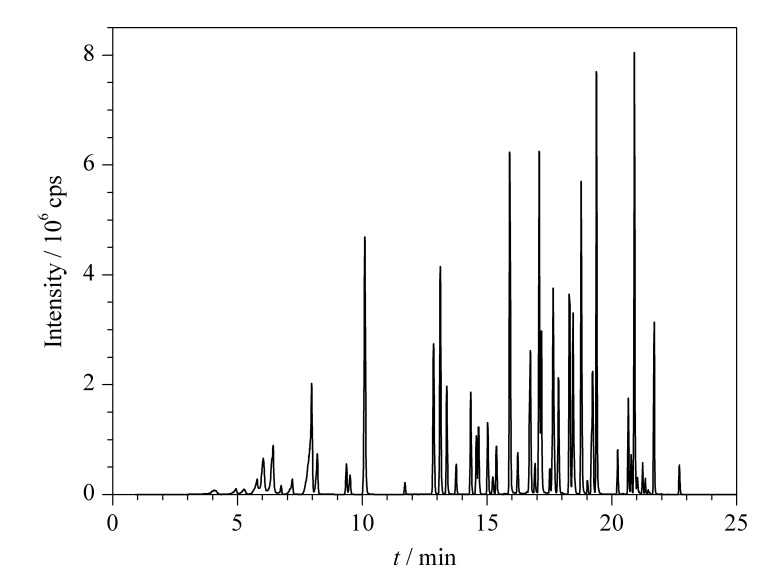
66种农药混合标准溶液(5.0 μg/L)的总离子流色谱图

### 2.2 MWCNTs-SAL的表征

按照1.3节方法制备MWCNTs-SAL, MWCNTs-SAL复合颗粒的光学照片和扫描电镜图如图2所示。MWCNTs-SAL复合颗粒的外观近似球形,直径约1 mm(图2a);由图2b可看出,MWCNTs被均匀地包围在SAL网络中,证明了MWCNTs-SAL复合颗粒的成功制备。

**图 2:**
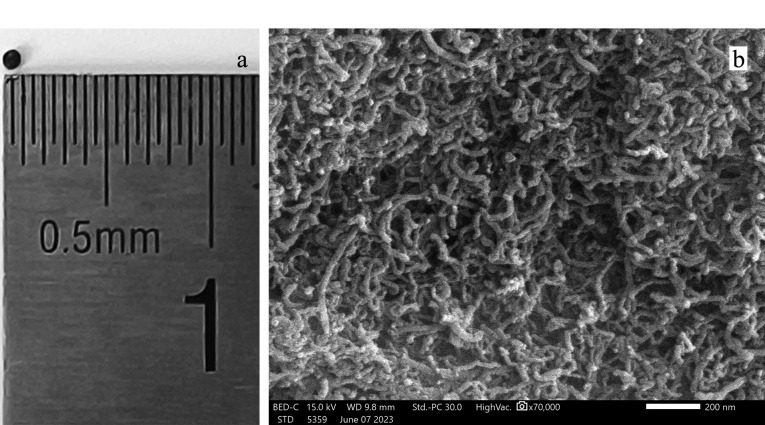
MWCNTs-SAL的(a)光学照片和(b)扫描电镜图

### 2.3 样品前处理条件的优化

#### 2.3.1 提取溶剂的选择

水产基质中含有大量的脂肪和蛋白质等干扰物质,将乙腈用作水产品中的农药提取溶剂时,不仅可以很好地提取目标农药,还能够有效沉淀蛋白质等干扰物质,因此乙腈是目前水产品农药提取过程中使用最多的有机溶剂^[5]^。本研究先使用乙腈对目标农药进行一次提取,之后加入乙酸进行二次提取,以提高酸性农药的提取效率,并进一步有效沉淀样品基质中的蛋白质。

#### 2.3.2 净化材料的选择

为了保护色谱柱和质谱仪,本研究在上机前采用推杆式震荡型净化柱对样品提取液进行净化。目前常用的净化材料有C_18_、PSA、中性氧化铝等。C_18_填料表面为疏水性的长链烷基基团,对提取液中的脂肪具有良好的吸附作用^[12]^; PSA填料表面含有两个氨基,可通过离子交换作用去除提取液中的脂肪酸和有机酸等干扰物质;HLB是亲水-亲脂平衡净化剂,其对脂肪、磷脂等干扰物质具有良好的吸附性。本文比较了不同净化材料对66种农药净化效果的影响,包括C_18_、PSA、HLB和MWCNTs-SAL(用量均为50 mg),实验结果详见图3。实验结果表明,当使用MWCNTs-SAL作为净化材料时,样品提取液的净化效果最好,大部分农药的回收率为70%~110%,因此本实验最终选择MWCNTs-SAL作为净化材料。

**图 3:**
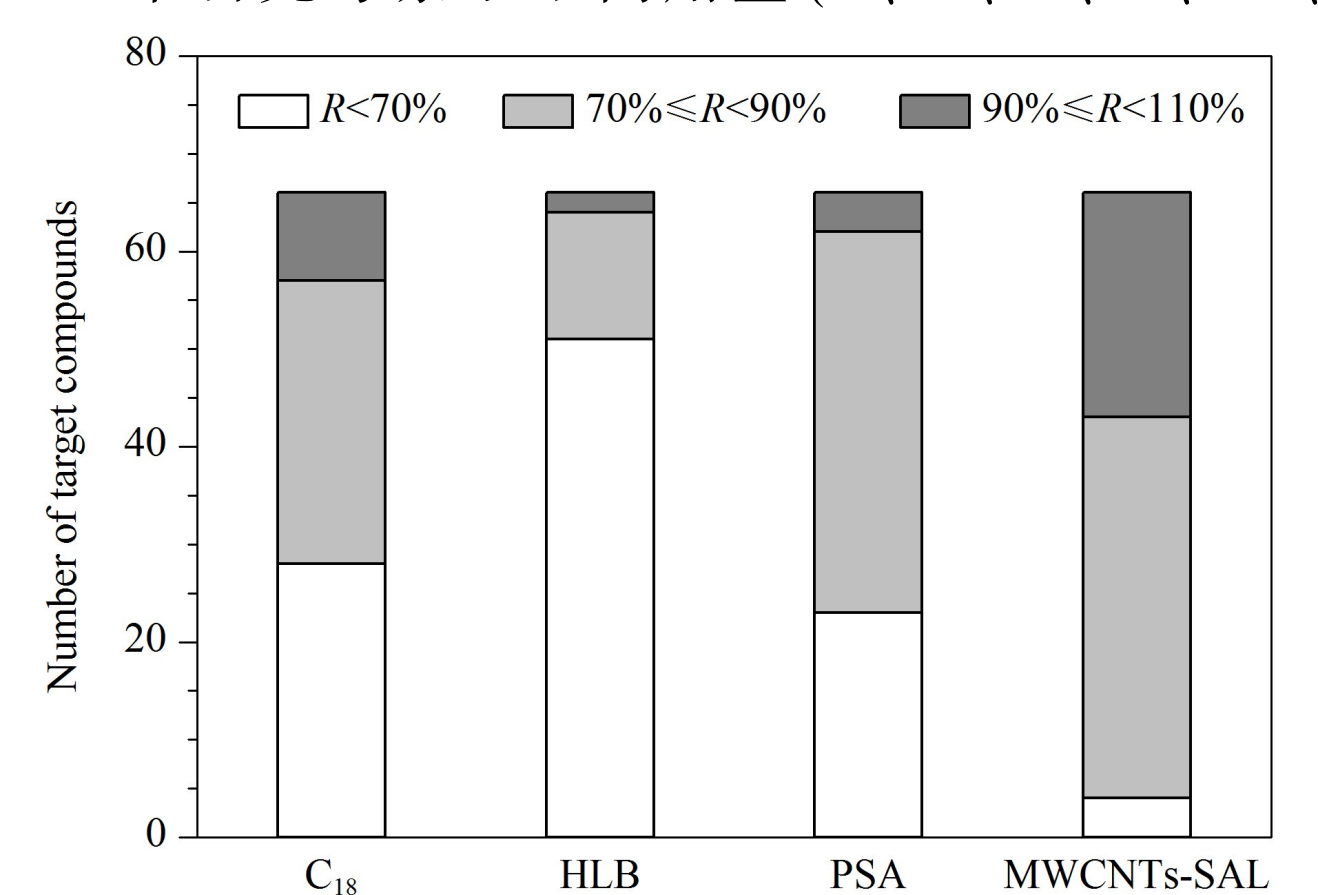
不同净化材料对66种农药净化效果的影响

#### 2.3.3 净化材料用量的优化

本研究考察了不同用量(0、20、50、75、100、125 mg)的MWCNTs-SAL对66种农药净化效果的影响,实验结果如图4a所示。结果表明,50 mg的MWCNTs-SAL对66种目标化合物的净化效果最为理想。水产品基质中的水分会对质谱分析造成干扰,本实验在净化过程中加入无水Na_2_SO_4_,以去除基质提取液中的水分,提高目标农药的提取效率。实验考察了不同用量(0、100、200、300、500 mg)的无水Na_2_SO_4_对66种目标化合物回收率的影响,结果如图4b所示。当使用100 mg无水Na_2_SO_4_时,回收率为90%~110%的目标化合物数量最多。因此,最终确定MWCNTs-SAL的用量为50 mg, Na_2_SO_4_的用量为100 mg。

**图 4:**
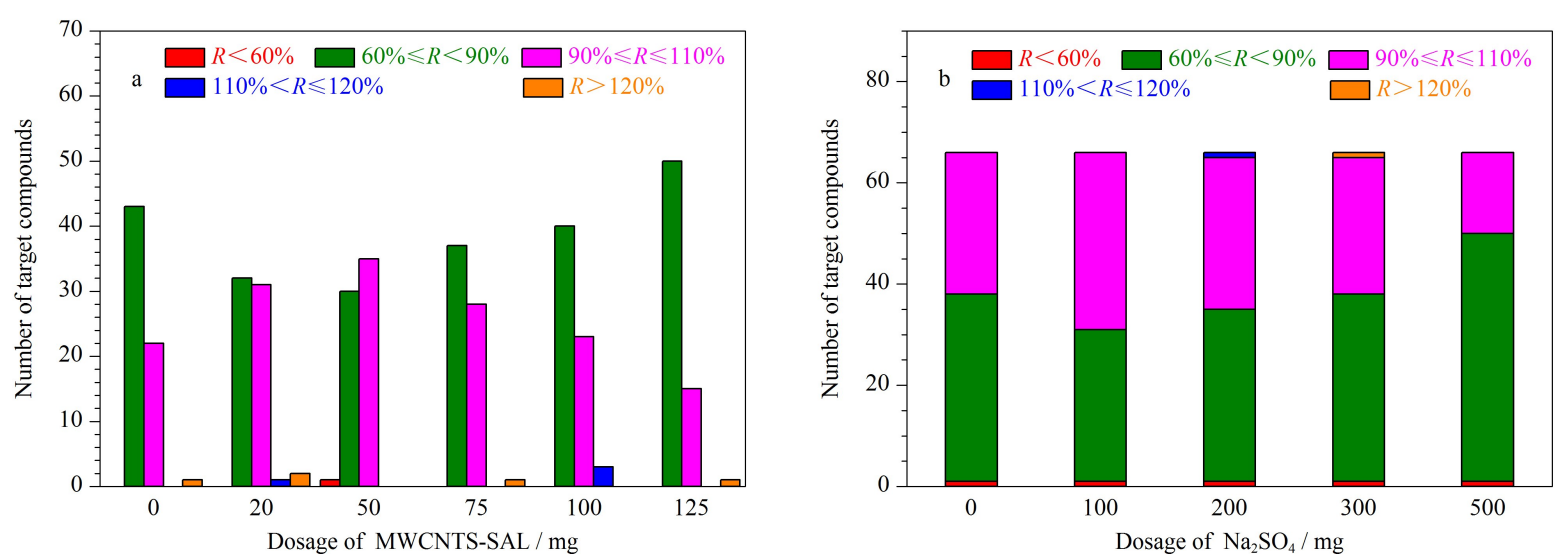
不同用量的(a)MWCNTs-SAL和(b)Na_2_SO_4_对66种农药净化效果的影响

### 2.4 基质效应

在质谱分析中,样品提取液中的干扰物质可能会影响目标化合物的质谱信号强度,产生基质效应(ME),从而影响目标化合物的定量结果。本研究对南美白对虾肌肉样品中66种农药的基质效应进行了评估。用空白样品基质提取液对66种农药的混合标准工作溶液进行稀释,配制成基质匹配混合标准溶液,根据ME=(Area_m_/Area_s_-1)×100%来计算基质效应;其中,Area_s_代表混合标准工作溶液的峰面积,Area_m_代表相同质量浓度的基质匹配混合标准溶液的峰面积。实验结果表明,乙螨唑的ME为-20.3%,表现为中等基质抑制效应;其余65种农药的ME为-20%~20%,表现为弱基质效应。为了提高定量分析结果的准确性,本研究采用基质匹配标准曲线对南美白对虾样品中的66种农药进行定量分析。

### 2.5 方法学验证

#### 2.5.1 线性方程、检出限和定量限

用空白样品基质提取液对66种农药的混合标准工作溶液进行稀释,配制成系列质量浓度(0.5、1、2、5、10、20、50 μg/L)的基质匹配混合标准溶液,上机检测。以目标化合物的质量浓度为横坐标(*x*, μg/L),峰面积为纵坐标(*y*),绘制基质匹配标准曲线。实验结果表明,66种农药在0.5~50 μg/L内线性关系良好,相关系数(*r*)均大于0.99;分别以信噪比(*S/N*)=3和*S/N*=10计算检出限(LOD)和定量限(LOQ),结果详见表3。66种农药的LOD和LOQ分别为0.5~1 μg/kg和1~2 μg/kg。以上实验结果说明,本方法具有较高的灵敏度。

**表 3:** 66种农药的检出限、定量限及其在3个加标水平下的加标回收率、日内和日间精密度

No.	Compound	LOD/ (μg/kg)	LOQ/ (μg/kg)	2 μg/kg					10 μg/kg					20 μg/kg					
				Recovery^*^/ %	Intra-day RSD^*^/%	Inter-day RSD^#^/%			Recovery^*^/ %	Intra-day RSD^*^/%	Inter-day RSD^#^/%			Recovery^*^/ %	Intra-day RSD^*^/%		Inter-day RSD^#^/%		
1	methamidophos	1	2	91.0	2.4	5.6			84.1	3.2	6.2			92.3		4.7		4.7	
2	acephate	0.5	2	79.9	2.3	6.2			88.4	2.2	4.1			82.9		6.7		6.7	
3	omethoate	0.5	2	83.9	1.4	7.4			89.1	2.3	5.4			90.8		5.5		8.1	
4	propamocarb	0.5	2	82.2	2.6	5.0			90.8	2.6	5.1			94.0		6.2		6.3	
5	aldicarb-sulfoxide	0.5	1	81.0	6.5	7.8			89.7	2.7	4.2			100.2		7.1		11.6	
6	aldicarb-sulfone	0.5	2	88.1	2.3	2.9			87.4	0.9	1.6			93.2		1.7		1.7	
7	carbendazim	0.5	2	81.8	1.5	2.2			85.7	0.7	1.2			91.6		1.3		2.1	
8	methomyl	0.5	2	84.6	1.9	2.2			89.3	1.4	3.7			98.3		2.6		6.5	
9	thiamethoxam	0.5	2	80.0	2.0	4.3			89.4	0.7	5.5			89.9		1.1		3.3	
10	imidacloprid	0.5	2	84.3	1.3	3.6			89.5	1.1	1.3			91.4		0.7		2.1	
11	clothianidin	1	2	83.6	2.0	3.2			89.2	0.8	1.6			90.5		0.8		1.8	
12	carbofuran-3-hydroxy	0.5	1	84.6	1.8	1.9			87.5	1.3	1.7			92.6		1.2		2.5	
13	acetamiprid	0.5	2	86.2	1.1	1.2			89.7	1.0	1.5			92.0		1.2		2.6	
14	dimethoate	0.5	2	85.4	0.9	1.9			90.1	1.2	1.1			91.3		0.9		1.2	
15	aldicarb	0.5	2	83.9	1.6	2.8			88.8	2.2	3.3			96.2		2.2		5.7	
16	dichlorvos	0.5	2	64.1	7.4	13.1			73.4	2.3	8.2			82.1		0.5		0.7	
17	thiophanatemethyl	0.5	1	78.5	1.3	3.8			80.1	1.0	2.5			85.5		1.5		4.2	
18	carbofuran	0.5	1	74.8	1.2	4.5			84.1	1.3	1.6			90.3		0.8		1.5	
19	fenthion-sulfoxide	0.5	1	81.7	1.7	3.3			85.2	1.7	1.9			90.4		3.3		4.5	
20	fenthion-sulfone	0.5	2	78.3	2.1	4.8			79.8	0.8	3.1			85.1		0.8		3.6	
21	phorate-sulfoxide	0.5	2	84.0	1.8	2.5			85.8	1.6	1.9			92.2		1.1		2.2	
22	phorate-sulfone	0.5	2	78.7	3.1	4.4			80.2	1.4	4.8			91.1		1.9		4.1	
23	isoprocarb	0.5	2	74.4	2.1	4.8			83.8	1.6	1.9			89.7		0.7		1.0	
24	pyrimethanil	0.5	2	75.5	4.0	4.7			84.1	2.1	2.5			89.3		1.6		1.6	
25	forchlorfenuron	0.5	2	71.5	1.7	5.7			78.7	1.1	1.4			88.6		1.1		5.0	
26	methidathion	0.5	2	80.3	1.6	2.9			81.0	1.5	2.9			87.5		0.7		2.0	
27	azoxystrobin	0.5	1	71.1	1.4	5.7			71.3	1.7	5.9			90.8		1.1		7.4	
28	dimethomorph	0.5	2	71.3	1.7	6.0			75.4	0.9	4.0			90.3		1.5		7.7	
29	malathion	0.5	2	71.7	1.6	6.9			74.2	1.6	6.1			82.1		1.9		5.1	
30	myclobutanil	0.5	2	70.9	3.7	7.9			77.3	2.1	2.8			81.3		1.6		5.2	
31	triazophos	0.5	2	71.4	2.4	5.7			71.0	1.1	5.3			81.0		0.6		5.2	
32	isazofos	0.5	2	71.9	1.5	5.2			74.1	2.0	5.5			79.7		1.4		6.2	
33	fipronil-desulfinyl	0.5	2	70.1	6.3	11.1			78.5	14.3	11.4			90.6		3.4		14.3	
34	epoxiconazole	0.5	2	72.4	3.7	5.1			76.5	2.7	5.0			94.4		2.0		8.7	
35	ethoprophos	0.5	1	75.0	3.2	6.5			83.3	2.8	3.4			89.0		2.9		2.7	
36	fenbuconazole	0.5	1	76.5	8.4	9.4			73.5	3.8	4.1			81.3		2.5		6.3	
37	fipronil	0.5	2	80.5	12.8	12.3			88.4	7.7	10.7			92.6		3.0		16.7	
38	flusilazole	0.5	2	70.5	3.9	9.1			75.3	1.6	3.4			92.0		2.2		9.8	
39	iprodione	0.5	1	72.9	10.3	10.8			80.2	4.9	5.7			101.6		1.6		15.0	
40	fipronil-sulfide	0.5	2	85.1	15.8	20.4			71.4	7.2	10.4			92.3		6.3		17.4	
41	prochloraz	0.5	2	86.0	4.5	9.8			81.7	3.7	5.7			101.2		6.8		6.5	
42	isofenphosmethyl	0.5	2	74.9	3.7	6.7			76.6	4.9	7.8			74.4		4.7		7.4	
43	isocarbophos	0.5	2	72.2	2.3	5.9			70.4	2.4	8.9			76.2		4.4		11.2	
44	fipronil-sulfone	0.5	2	70.7	3.6	6.0			79.3	11.0	11.0			97.5		3.1		18.7	
45	tebuconazole	0.5	2	69.2	3.2	6.6			71.4	1.3	3.6			86.1		1.3		4.0	
46	fenthion	0.5	2	74.5	4.7	7.4			73.8	2.7	6.4			76.1		1.3		8.4	
47	famoxadone	0.5	2	72.9	6.2	8.5			77.1	2.0	2.6			92.2		1.6		14.0	
48	phoxim	0.5	2	70.8	4.7	6.1			74.4	8.7	7.9			74.0		1.8		11.0	
49	pyraclostrobin	0.5	2	72.9	3.7	5.9			71.1	1.6	5.9			93.3		3.3		15.0	
50	hexaconazole	0.5	2	71.8	4.5	7.7			74.7	2.3	2.8			79.9		1.2		6.3	
51	phorate	0.5	2	74.6	2.8	6.4			70.9	4.7	6.6			88.7		2.6		8.5	
52	difenoconazole	0.5	2	70.4	2.4	7.7			71.0	1.3	6.1			75.5		1.3		8.2	
53	(E)-diniconazole	0.5	1	78.3	6.8	8.4			71.0	1.5	4.8			94.9		2.2		10.2	
54	emamectin benzoate	0.5	2	69.9	10.7	10.7			70.3	14.8	13.0			87.8		3.2		15.7	
55	trifloxystrobin	0.5	1	72.0	8.3	7.9			73.3	8.7	7.0			92.8		1.4		15.5	
56	tolfenpyrad	0.5	2	80.4	5.3	5.3			78.6	2.0	5.4			107.3		1.9		14.9	
57	fenpropathrin	0.5	1	72.2	3.3	7.0			74.4	1.9	4.6			91.8		2.1		13.5	
58	chlorpyrifos	0.5	1	71.4	4.1	7.8			77.0	2.8	4.9			80.1		4.7		11.5	
59	pendimethalin	0.5	2	72.1	2.2	7.1			73.3	2.5	4.5			92.9		1.6		14.1	
60	etoxazole	0.5	2	71.5	3.8	6.1			71.2	2.7	5.5			92.7		2.0		14.4	
61	propargite	0.5	2	75.4	6.9	6.0			73.5	3.6	15.9			98.4		2.9		16.4	
62	(E)-fenpyroximate	0.5	2	73.1	3.7	7.6			77.4	3.0	3.7			93.5		6.4		18.9	
63	esfenvalerate	0.5	1	83.3	7.5	7.6			81.2	4.4	7.9			96.1		7.5		12.6	
64	fenvalerate	0.5	2	72.3	12.0	14.2			76.0	6.2	9.8			101.6		6.8		7.9	
65	pyridaben	0.5	2	73.5	11.0	12.3			81.4	10.4	18.0			94.4		2.9		11.9	
66	bifenthrin	0.5	2	75.0	7.9	8.4			71.7	6.0	9.4			94.4		12.8		12.6	

* *n*=6; # *n*=3.

#### 2.5.2 回收率和精密度

按照1.5节方法对南美白对虾空白样品进行前处理后,分别添加低、中、高3个水平(2、10、20 μg/kg)的混合标准工作溶液,平行测定6次,计算加标回收率(*n*=6);计算回收率的相对标准偏差,得到日内精密度(*n*=6);连续测定3 d,计算日间精密度(*n*=3),结果见表3。实验结果表明,在3个加标水平下,66种农药的加标回收率为64.1%~107.3%,日内及日间精密度均小于20.4%,说明该方法准确度高,重复性好,能够满足水产品中农药残留的检测需求。

### 2.6 实际样品的检测

利用本文所建方法对从北京市市场随机抽取的15份南美白对虾实际样品进行检测,结果表明,所有样品中均未检出66种目标农药残留。

## 3 结论

本研究基于MWCNTs-SAL复合净化材料和HPLC-QTRAP-MS/MS技术,建立了水产品中66种农药残留的快速分析方法。实验所制备的MWCNTs-SAL复合净化材料具有优异的净化性能,可以简便、快速地对水产品基质提取液进行净化处理。该方法方便快捷,经济高效,灵敏度高,重复性好,为药物残留检测方法开发、食用水产品中农药污染情况调查以及农药残留风险评估等方面提供了理论和技术支持。

## References

[1] Eunjung K, Sihyun P, Hyunjin P, et al. Molecules, 2021, 26(9): 2575

[2] Wang S Y, Yang G X, Tang Y Y, et al. Foods, 2023, 12(6): 1131

[3] Wang C X, Huang F, Wang M, et al. Chinese Journal of Chromatography, 2013, 31(3): 206 10.3724/sp.j.1123.2012.11010 23785991

[4] Zhang Y, Qiao H, Chen C, et al. Food Chem, 2016, 192: 612 26304390 10.1016/j.foodchem.2015.07.035

[5] Guo Y G, Zhang J, Xu J, et al. J Agric Food Chem, 2021, 69(25): 7199 10.1021/acs.jafc.0c08040 34142545

[6] Gosetti F, Mazzucco E, Zampieri D, et al. J Chromatogr A, 2010, 1217(25): 3929 10.1016/j.chroma.2009.11.060 20004403

[7] Qin Y H, Huang B Y, Zhang J R, et al. J Sep Sci, 2016, 39(9): 1757 10.1002/jssc.201501401 26968118

[8] Ma L L, Zhao L W, Wang J Q, et al. J Chromatogr Sci, 2020, 58(2): 109 10.1093/chromsci/bmz081 31711217

[9] Zou N, Chen R H, Qin Y H, et al. J Sep Sci, 2016, 39(18): 3638 10.1002/jssc.201600614 27440123

[10] Song L, Zeng W B, Li A, et al. Food Control, 2022, 131: 108436

[11] Meng Z J, Li Q, Cong J H, et al. Foods, 2021, 10(7): 1651 10.3390/foods10071651 PMC8305287 34359521

[12] Mu S H, Wang C Y, Liu H, et al. Biomed Chromatogr, 2021, 35(10): e5176 10.1002/bmc.5176 33990966

[13] Zhou R D, Mu S H, Feng T, et al. J Food Compos Anal, 2022, 109: 104506

[14] Dong X Q, Lan T, Tian X, et al. J Environ Sci Heal B, 2021, 56(8): 771 10.1080/03601234.2021.1944962 34190035

[15] Wang J, Wu T, Wang X Q, et al. Chinese Journal of Chromatography, 2023, 41(4): 330 10.3724/SP.J.1123.2022.08005 PMC10071351 37005920

[16] Tu X T, Yang H B, Guo F, et al. Chinese Journal of Analytical Chemistry, 2021, 49(4): 528

